# Supplementary material for: A theory of oligogenic adaptation of a quantitative trait
Source: Genetics. 2023 Aug 8;225(2):iyad139. doi: 10.1093/genetics/iyad139 (PMC10550320; doi:10.1093/genetics/iyad139)
Supplement: iyad139_Supplementary_Data [file iyad139_supplementary_data.pdf]

S1 Supplemental Material
A theory of oligogenic adaptation of a quantitative trait

Ilse Höllinger, Benjamin Wölfl and Joachim Hermisson

S1 Mathematical Supplement

This supplement describes the details of the mathematical model and methods used to derive the analytical results of the article. It also demonstrates how the methods generalize to a more general class of selection models.

S1.1 Quantitative trait model

Ecological assumptions Consider a panmictic population of N_e haploids. Selection acts on a quantitative trait Z. Let

$$w(Z,t) = \sum_{j=0}^{\infty} w_j(t) Z^j \tag{S1.1}$$

be the Malthusian (logarithmic) fitness function written as a Taylor series around $Z = 0$ with coefficients $w_j(t)$. We are interested in the scenario where an equilibrium population adapts to a rapid change in the environment. We thus assume that fitness is explicitly time-dependent, but leave the time dependence mostly implicit in our notation below, *i.e.*, $w(Z,t) \equiv w(Z)$ and $w_j(t) \equiv w_j$. The standard Gaussian model of stabilizing selection (Bürger, 2000) with a shift in the optimum (that is used throughout in the *main text*) is a particular example. It corresponds to the quadratic Malthusian fitness function

$$w(Z,t) = -\frac{\sigma(t)}{2}\Big(Z - Z_\mathrm{opt}(t)\Big)^2 \tag{S1.2}$$

with $Z_\mathrm{opt}(0) = Z^0_\mathrm{opt}$ and $Z_\mathrm{opt}(t \geq 0) > Z^0_\mathrm{opt}$ and fits the general scheme (S1.1) with $w_0(t) = -(\sigma(t)/2)Z_\mathrm{opt}(t)^2$, $w_1(t) = \sigma(t)Z_\mathrm{opt}(t)$, and $w_2(t) = -\sigma(t)/2$. The corresponding Wrightian fitness (for discrete time) follows as $W(Z,t) = \exp[w(Z,t)]$.

Genetic assumptions We assume the standard additive model of a quantitative trait (Falconer and Mackay, 1996), which reads

$$Z = \sum_{i=1}^{L} \gamma_i \eta_i \quad \text{or} \quad Z = \sum_{i=1}^{L} \gamma_i (\eta_i + \eta_i') \tag{S1.3}$$

for the haploid or diploid case, respectively. The $\eta_i, \eta_i' \in 0, 1$ indicate the allelic state at locus i. All L loci are bi-allelic, where the effect of allele a_i (corresponding to $\eta_i = 0$ or $\eta_i' = 0$) on the phenotype is normalized to 0 and the effect of the "plus" allele A_i

(corresponding to $\eta_i = 1$ or $\eta_i' = 1$) is $\gamma_i > 0$. We will later assume equal effect sizes at all loci ($\gamma_i \equiv \gamma$) for the derivation of our main result, but allow for general effect sizes as long as possible. Although the results extend to co-dominant diploids, we will focus on haploids throughout. We denote the frequency of plus alleles A_i with p_i and the frequency of the alternative a_i alleles as $q_i = 1 - p_i$. New mutations from a_i to A_i occur at rate μ_i per generation and back mutations at rate ν_i. Denote the mean fitness as $\bar{w}$ and the marginal fitnesses of alleles A_i and a_i as $\bar{w}_{A_i}$ and $\bar{w}_{a_i}$, respectively. The evolutionary dynamics in continuous time read (Bürger, 2000)

$$\dot{p}_i = p_i(1-p_i)(\bar{w}_{A_i} - \bar{w}_{a_i}) + \mu_i(1-p_i) - \nu_i p_i, \tag{S1.4}$$

where we assume Hardy-Weinberg equilibrium in the case of diploids. In our general model that is used in the individual-based simulations, loci may be linked with recombination rate r between neighboring loci on a linear chromosome. For our analytical treatment (and the Wright-Fisher simulations), however, we assume linkage equilibrium (LE) among all loci. The individual-based and Wright-Fisher simulations are described in the *Computational Supplement* deposited on *Dryad* (Höllinger et al., 2023). For the analytical treatment we can then expand the selection part of the single-locus dynamics (S1.4) in terms of the locus effects as shown in the following Theorem 1 (all proofs are deferred to the section S1.3 Proofs).

Theorem 1 Assuming LE, the selection dynamics can be written as a Taylor series of the locus effects γ_i around 0 as

$$\dot{p}_i = p_i(1-p_i)(\bar{w}_{A_i} - \bar{w}_{a_i}) = p_i(1-p_i) \sum_{m=1}^{\infty} \frac{f_m(Z)}{m!} A_m(p_i)\, {\gamma_i}^m, \tag{S1.5a}$$

where

$$f_m(Z) = \left\langle \frac{\partial^m}{\partial Z^m} w(Z) \right\rangle = \sum_{j=m}^{\infty} \frac{j!\, w_j}{(j-m)!} \langle Z^{j-m} \rangle \tag{S1.5b}$$

is the mth derivative of the population-averaged (denoted $\langle \dots \rangle$) fitness function and

$$A_m(p_i) = 1 - \sum_{k=1}^{m-1} {p_i}^k \sum_{\ell=1}^{k+1} \binom{k+1}{\ell} (-1)^{\ell} \ell^m . \tag{S1.5c}$$

Find the proof in Proof of Theorem 1.

Remark 1 The leading orders of the single-locus dynamics read

$$\dot{p}_i = p_i(1-p_i)\Big(\gamma_i f_1(Z) + \frac{{\gamma_i}^2}{2!} f_2(Z)(1-2p_i) + \frac{{\gamma_i}^3}{3!} f_3(Z)\big(1 - 6p_i(1-p_i)\big) + \frac{{\gamma_i}^4}{4!} f_4(Z)(1-2p_i)\big(1-12p_i(1-p_i)\big) + \mathcal{O}[{\gamma_i}^5] \Big).$$

For the quadratic Malthusian fitness function (S1.2), this results in the familiar expression (Barton, 1986)

$$\dot{p} = p_i(1-p_i)\Big(\sigma(t)\gamma_i(Z_{\mathrm{opt}}(t)-\bar{Z}) + \frac{\sigma(t)}{2}{\gamma_i}^2(2p_i-1)\Big) \tag{S1.6}$$

with a first-order selection term that depends only on the distance of the trait mean from the optimum and a second-order term giving rise to disruptive selection on the allele frequencies. In general, the derivatives $f_k(Z)$ of the fitness function can depend on all moments of the trait. They depend explicitly (via time-dependent selection) and implicitly (via changes in the trait moments) on time and change as evolution proceeds, $f_k(Z) \equiv f_k(Z,t)$.

Directional selection model

For our further analytical progress, we assume that the single-locus dynamics can be approximated as

$$\dot{p}_i \approx p_i(1-p_i)s(\gamma_i, Z, t) + \mu_i(1-p_i) - \nu_i p_i, \tag{S1.7}$$

where the selection coefficient $s(\gamma_i, Z, t)$ depends on the locus effect γ_i and the phenotype distribution (moments of Z), but not directly on the allele frequencies, p_i. This means that selection at all loci is essentially directional, but for a common scaling function s that summarizes all interactions, both within and between loci. Using Theorem 1, we identify three scenarios where this approximation applies, which are all relevant in our context.

1. Firstly, the approximation generally holds for loci with sufficiently small allele frequency p_i near 0. In this case, $A_m(p_i\!=\!0) = 1$ (also for $p_i \approx 0$) in (S1.5c) and

   $$s(\gamma_i, Z, t) := \sum_{m=1}^{\infty} \frac{{\gamma_i}^m}{m!}\, f_m(Z,t)\,.$$

   For quadratic selection (S1.2) with the trait mean at the optimum (*i.e.*, before the environmental change), in particular, we have (using S1.6)

   $$\dot{p}_i \approx -p_i(1-p_i)\frac{\sigma(t)}{2}{\gamma_i}^2 \approx -p_i\frac{\sigma(t)}{2}{\gamma_i}^2\,.$$

   With a scaled selection parameter $S_i = N_{\mathrm{e}}\sigma{\gamma_i}^2$ acting on rare alleles at locus i, the density in the standing genetic variation is approximately

   $$\rho(p_i) \sim {p_i}^{\Theta_i - 1}\exp[-S_i \cdot p_i]$$

   with $\Theta_i = 2N_{\mathrm{e}}\mu_i$ (Hermisson and Pennings, 2005). For $S_i \gg \Theta_i$, almost all alleles have starting frequencies of $p_i < 0.1$. For our results below, the distribution of alleles in the standing variation *that are destined for later establishment* is even

more relevant. If the optimal trait value shifts to a new value $Z_{\text{opt}} \gg \bar{Z}$, this is given by (Hermisson and Pennings, 2005)

$$\tilde{\rho}(p_i) \sim p_i^{\Theta_i - 1} \exp[-S_i \cdot p_i] \Big(1 - \exp[-2S_i(Z_{\text{opt}} - \bar{Z})/\gamma_i \cdot p_i)]\Big). \tag{S1.8}$$

If we restrict our interest to $S_i \geq 10$ (throughout the *main text*) and $\Theta_i \lesssim 1$ (violated for cases with $L = 10$ and $\Theta_{\text{bg}} = 100$, *e.g.,* Fig. 3 of the *main text*), a large majority of alleles has low starting frequency (*cf.* Fig. S1.1).

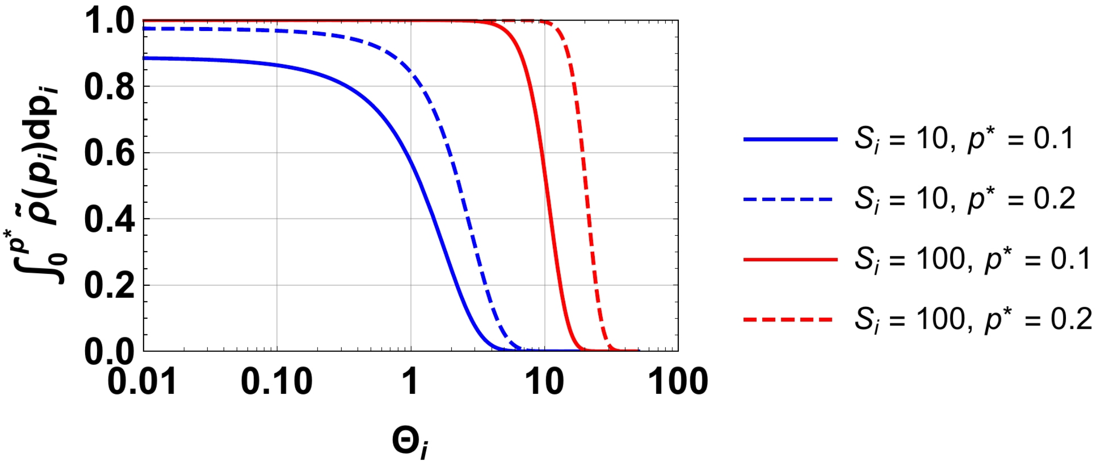

Figure S1.1: Cumulative density for $\tilde{\rho}(p_i)$ following Eq. (S1.8) with $(Z_{\text{opt}} - \bar{Z})/\gamma_i = 3$.

2. Secondly, for larger allele frequencies, the directional selection model (S1.7) still applies with $s(\gamma_i, Z, t) := \gamma_i f_1(Z, t)$, whenever the selection dynamics are dominated by the first-order term of the expansion in γ_i,

$$\gamma_i f_1(Z, t) \gg \frac{\gamma_i^k}{k!} f_k(Z, t), \quad \forall\, i,\ \forall\, k > 1\,.$$

For quadratic selection (S1.6), in particular, this leads to

$$\dot{p}_i = p_i(1 - p_i)\sigma(t)\gamma_i(Z_{\text{opt}}(t) - \bar{Z})\,,$$

which has previously been used to approximate the adaptation dynamics for a quantitative trait (Jain and Stephan, 2017). The approximation applies after the environmental change as long as the trait mean is sufficiently far from the optimum, such that single alleles do not yet overshoot it,

$$\gamma_i \lesssim (Z_{\text{opt}}(t) - \bar{Z}), \quad \forall\, i.$$

Note that this condition does not depend on the selection strength $\sigma(t)$ on the trait. It also does not imply that the *distribution of trait values* Z in the population does not overshoot the optimum. For a polygenic trait, this will frequently be the case.

3. Third, the approximation can also be used for high allele frequencies p_i near 1. We derive $A_m(p_i\!=\!1) = -(-1)^m$ (see Proof of Proposition 1.1) and

$$s(\gamma_i, Z, t) := -\sum_{m=1}^{\infty} \frac{(-\gamma_i)^m}{m!} f_m(Z,t)\,.$$

   It is convenient to express the dynamical equations in terms of the frequencies $q_i = 1 - p_i$ of the alternative allele a_i in this case. For quadratic selection with the mean at the optimum, this reads (with $\dot{q}_i = -\dot{p}_i$),

$$\dot{q}_i = -q_i(1-q_i)\frac{\sigma(t)}{2}{\gamma_i}^2 \approx -q_i\frac{\sigma(t)}{2}{\gamma_i}^2\,, \tag{S1.9}$$

   entirely analogous to the case of small p_i above.

These observations show that the deterministic dynamics of mutant alleles of an additive trait under stabilizing selection that subsequently experiences a phase of directional selection can generally be described by a directional selection model of the form (S1.7), both before and after the environmental change as long as we are not too close to the *new* optimum. In particular, this holds for a trait under Gaussian stabilizing selection that adapts to a shift in the optimum. We now extend the model to include genetic drift, following a procedure previously described in Höllinger et al. (2019) (see also Joyce and Tavaré 1987; Hermisson and Pfaffelhuber 2008).

Stochastic dynamics: Moran model and Yule approximation

We want to construct the stochastic dynamics of mutant alleles that conforms to the deterministic directional selection model (S1.7). This is most easily done in terms of a continuous-time Moran model (Moran, 1958), where individuals in the population give birth at a specific rate and each newborn replaces some other individual which is randomly selected to die. New mutant individuals are generated by two processes: new mutation and existing mutants giving birth and replacing a wildtype individual (a split of mutant lineages). In a population of haploid size N_e, new A_i mutations occur at a rate $N_\mathrm{e}\mu_i(1-p_i)$ per generation. Splits generate new mutants that replace wildtype individuals at rate $(1+s(\gamma_i,Z,t))(1-p_i)$ per mutant individual and generation. The number of mutants can also decrease if a splitting wildtype replaces a mutant, leading to an effective death rate of $(1-p_i)$ per generation. Note that we do not include events which keep the number of mutant alleles unaltered (*e.g.*, mutants replacing other mutants). Mathematically, the dynamics of the A_i mutants are given by a *birth-death process with immigration* (where "immigration" corresponds to the biological process of new mutation).

We are interested in the joint distribution of mutant alleles at all loci at some time of observation during or after rapid adaptive evolution. Consider a mutation A_i that appears either prior to the adaptive phase (standing genetic variation) or during this phase (new mutation). This mutation is relevant for the joint distribution if (and only if) descendants of the mutation still segregate in the population at the time of observation.

We thus only need to account for mutations that found *immortal lines of descent* (*i.e.*, immortal at least until the sampling time). We thus describe a coalescent genealogy, but forward in time and across multiple loci simultaneously. Mathematically, this leads to a *Yule process*, where we decide directly at birth whether a lineage survives or dies and include it only when it is destined to survive.

Yule process Stochastic effects are particularly important as long as the frequencies of alleles that later contribute to the adaptive architecture are small, $p_i \ll 1$. To describe this early stochastic phase, denote the survival (or *establishment*) probability of a single mutant lineage at locus i and time t as $p_{\mathrm{est},i}(t)$. A time-inhomogeneous, multitype Yule process is now defined by two types of events and their respective rates:

1. New immortal lines (across all loci) are created by mutation at rate

$$p_{\mathrm{mut},i}(t) = N_{\mathrm{e}}\mu_i(1-p_i)p_{\mathrm{est},i}(t) \approx N_{\mathrm{e}}\mu_i p_{\mathrm{est},i}(t).$$

2. Existing lines split at rates $p_{\mathrm{split},i}(t)$, which can be determined as follows. We are only interested in splits in the underlying Moran model that lead to *two* immortal lines, $\sim (p_{\mathrm{est},i}(t))^2$, where we already know that at least one descendant lineage is immortal (because we only consider splits of immortal lines in the first place). This leads to

$$p_{\mathrm{split},i}(t) = \frac{(1+s(\gamma_i,Z,t))(1-p_i)(p_{\mathrm{est},i}(t))^2}{(p_{\mathrm{est},i}(t))^2 + 2p_{\mathrm{est},i}(t)(1-p_{\mathrm{est},i}(t))} \approx \frac{p_{\mathrm{est},i}(t)}{2}$$

assuming that both $p_{\mathrm{est},i}(t) \ll 1$ and $s(\gamma_i,Z,t) \ll 1$.

In general, whether a new lineage (introduced at t') establishes (or not) depends on the sequence of birth and death events of the underlying Moran process and thus on the selection coefficients $s(\gamma_i,Z,t)$ for all $t \geq t'$. Effectively, however, the fate is already decided during a short time window while the number of descendant mutant lineages is small. If we ignore any changes in the strength of directional selection during this time, we can approximate $p_{\mathrm{est},i}(t) \approx p_{\mathrm{est}}(s(\gamma_i,Z,t))$. Note that exactly the same rates for splits and new mutation hold for rare a_i mutants as long as their frequencies are small, $q_i \ll 1$.

Equal loci Consider now a model with equal effect sizes at all loci, $\gamma_i \equiv \gamma$. Then also the selection coefficients $s(\gamma_i,Z,t) \equiv s(Z,t)$ at any given time are the same at all loci, and the same holds for the establishment probability, $p_{\mathrm{est},i}(t) \equiv p_{\mathrm{est}}(t) \approx p_{\mathrm{est}}(s(Z,t))$. Establishment thus acts as a common factor for all events across all loci. If we are not interested in the absolute speed of the process, but rather in the relative probabilities of events, we can ignore this factor (or eliminate it in a time transform), leading to a time-homogeneous process with

$$p_{\mathrm{mut},i} = \frac{\Theta_i}{2}, \quad p_{\mathrm{split},i} = \frac{1}{2}, \tag{S1.10}$$

where $\Theta_i = 2N_e\mu_i$ is the population mutation parameter. For this process, Höllinger et al. (2019) proved the following: Consider d loci with equal effect and let k_i be the number of mutant lineages at locus $i = 1, \ldots, d$. We start the process with no mutants ($k_i \equiv 0$) and stop it whenever the number of mutants at one of the loci (*e.g.*, locus 1) reaches some number $k_1 = n$. Then the distribution of *ratios* $x_i = k_i/n$ of mutants at the ith locus relative to the first locus converges in the limit where $n \to \infty$ to the so-called *inverted Dirichlet distribution* with $x_1 = 1$ and

$$\mathrm{P}_{\mathrm{inDir}}[\{x_i\}_{i\geq 2}|\mathbf{\Theta}] = \frac{1}{\mathrm{B}[\mathbf{\Theta}]} \Big(\prod_{j=1}^{d} {x_j}^{\Theta_j - 1}\Big)\Big(\sum_{j=1}^{d} x_j\Big)^{-\sum_{j=1}^{d}\Theta_j} \tag{S1.11}$$

where the vector $\mathbf{\Theta} = (\Theta_1, \ldots, \Theta_d)$ summarizes the mutation rates and $\mathrm{B}[\mathbf{\Theta}]$ is the multivariate beta function, which can be expressed in terms of gamma functions as

$$\mathrm{B}[\mathbf{\Theta}] = \frac{\prod_{i=1}^{d}\Gamma(\Theta_i)}{\Gamma(\sum_{i=1}^{d}\Theta_i)}\,. \tag{S1.12}$$

Note that $x_i = k_i/n = p_i/p_1$ can also be understood as a ratio of allele frequencies. In a sufficiently large population, and if selection is not too weak, this limit distribution for frequency ratios is (approximately) reached already by the end of the stochastic phase, while individual mutant frequencies p_i or q_i are still small.

Deterministic dynamics

While the frequency of (initially rare) a_i mutants stays small throughout the adaptive phase, the frequency p_i of positively selected A_i mutants grows and the Yule-process approximation no longer holds once $p_i \gg 0$. Once this is the case, however, we can largely ignore the effects of genetic drift and also of mutation, at least on the short time-scale of rapid adaptation. We can then use the deterministic directional selection model (S1.7) derived above to describe the further dynamics. It is convenient to apply a transformation of frequencies p_i to odds $u_i := p_i/(1-p_i)$ to obtain a simple model of time-dependent exponential growth for the u_i,

$$\dot{u}_i = \frac{\dot{p}_i}{(1-p_i)^2} = u_i s(\gamma_i, Z, t). \tag{S1.13}$$

The u_i are unbounded variables and run from 0 to ∞. Their dynamics are still coupled via $s(\gamma_i, Z, t)$, which depends on the phenotype distribution. For the quadratic selection model S1.6, in particular, we obtain

$$\dot{u}_i = u_i \sigma(0)\gamma_i(Z_{\mathrm{opt}} - \bar{Z}). \tag{S1.14}$$

Assume now, once again, that locus effects are equal, $\gamma_i = \gamma_1 \equiv \gamma$. We then find that

$$\dot{\left(\frac{u_i}{u_1}\right)} = \frac{\dot{u}_i u_1 - u_i \dot{u}_1}{{u_1}^2} = \frac{u_i}{u_1}\big(s(\gamma_i, Z, t) - s(\gamma_1, Z, t)\big) = 0\,,$$

i.e., the *odds ratios* are maintained under these dynamics, for any selection strength and functional form or time-dependence of $s(\gamma_i, Z, t)$. Since $u_i/u_1 \approx p_i/p_1 = x_i$ as long as allele frequencies p_i are small, we conclude that the joint distribution of odds ratios of loci with equal effect approximately follows the inverted Dirichlet distribution (S1.11), also after the early stochastic phase.

The joint allele frequency distribution We can now use this information to derive the joint distribution of allele frequencies at the time of sampling. Assume that there are d loci of equal effect, γ, and sampling occurs when their joint contribution to the mean trait is γc_Z. Then

$$\sum_{i=1}^{d} p_i = \sum_{i=1}^{d} \frac{u_i}{u_i + 1} = c_Z \,. \tag{S1.15}$$

with $c_Z \in [0, d]$. The odds ratios can be expressed in terms of allele frequencies as

$$x_i = \frac{u_i}{u_1} = \frac{p_i(1-p_1)}{(1-p_i)p_1} = \frac{p_i(1+\sum_{k=2}^{d} p_k - c_Z)}{(1-p_i)(c_Z - \sum_{k=2}^{d} p_k)}. \tag{S1.16}$$

The Jacobian of this transformation reads

$$\begin{aligned} J_{ij} = \frac{\partial x_i}{\partial p_j} &= \frac{p_i}{(1-p_i)(c_Z - \sum_{k=2}^{d} p_k)^2} + \delta_{i,j} \frac{1+\sum_{k=2}^{d} p_k - c_Z}{(1-p_i)^2(c_Z - \sum_{k=2}^{d} p_k)} \\ &= \frac{p_i(1-p_i) + \delta_{ij}\, p_1(1-p_1)}{(1-p_i)^2 {p_1}^2} \end{aligned} \tag{S1.17}$$

with determinant

$$\begin{aligned} \mathrm{Det}[\mathbf{J}] &= \prod_{i=2}^{d} \frac{1}{(1-p_i)^2} \left(\frac{1+\sum_{i=2}^{d} p_i - c_Z}{c_Z - \sum_{i=2}^{d} p_i}\right)^{d-1} \left(1 + \frac{\sum_{i=2}^{d} p_i(1-p_i)}{(1+\sum_{i=2}^{d} p_i - c_Z)(c_Z - \sum_{i=2}^{d} p_i)}\right) \\ &= \prod_{i=2}^{d} \frac{1}{(1-p_i)^2} \left(\frac{1-p_1}{p_1}\right)^{d-1} \left(1 + \frac{\sum_{i=2}^{d} p_i(1-p_i)}{p_1(1-p_1)}\right) \\ &= \prod_{i=1}^{d} \frac{1}{(1-p_i)^2} \left(\frac{1-p_1}{p_1}\right)^{d} \sum_{i=1}^{d} p_i(1-p_i)\,. \end{aligned}$$

The joint distribution of the p_i at the stopping condition then follows from (S1.11) as

$$\begin{aligned} \mathrm{P}[\{p_i\}|\mathbf{\Theta}, c_Z] &= \frac{\mathrm{Det}[\mathbf{J}]}{\mathrm{B}[\mathbf{\Theta}]} \prod_{i=1}^{d} \left(\frac{p_i(1-p_1)}{(1-p_i)p_1}\right)^{\Theta_i - 1} \left(\sum_{i=1}^{d} \frac{p_i(1-p_1)}{(1-p_i)p_1}\right)^{-\sum_{i=1}^{d}\Theta_i} \\ &= \frac{1}{\mathrm{B}[\mathbf{\Theta}]} \prod_{i=1}^{d} \frac{{p_i}^{\Theta_i - 1}}{(1-p_i)^{\Theta_i+1}} \left(\sum_{i=1}^{d} p_i(1-p_i)\right) \left(\sum_{i=1}^{d} \frac{p_i}{1-p_i}\right)^{-\sum_{i=1}^{d}\Theta_i} \end{aligned} \tag{S1.18}$$

with the constraint (S1.15) as boundary condition. Due to this constraint, the distribution is $(d-1)$-dimensional. For given frequencies at $d-1$ loci, the frequency at the last locus follows, *e.g.*, $p_1 = c_Z - \sum_{k\geq 2} p_k$. This way, also the dependence on the sampling condition c_Z enters. For two loci, in particular, the one-dimensional distribution for the frequency at the second locus reads ($p_2 \equiv p$)

$$\begin{aligned}\mathrm{P}[p|c_Z] =& \frac{\Gamma(\Theta_1+\Theta_2)}{\Gamma(\Theta_1)\Gamma(\Theta_2)} \frac{p^{\Theta_2-1}}{(1-p)^{\Theta_2+1}} \left(1+\frac{p(1-p)}{(c_Z-p)(1+p-c_Z)}\right) \\ &\cdot \left(1+\frac{1+p-c_Z}{c_Z-p}\frac{p}{1-p}\right)^{-\Theta_1} \left(\frac{c_Z-p}{1+p-c_Z}+\frac{p}{1-p}\right)^{-\Theta_2} \\ =& \frac{\Gamma(\Theta_1+\Theta_2)}{\Gamma(\Theta_1)\Gamma(\Theta_2)} \frac{p(1-p)+(c_Z-p)(1+p-c_Z)}{\big(c_Z-2p(c_Z-p)\big)^{\Theta_1+\Theta_2}} \\ &\cdot \big((c_Z-p)(1-p)\big)^{\Theta_1-1}\big((1+p-c_Z)p\big)^{\Theta_2-1}. \end{aligned} \tag{S1.19}$$

S1.2 The architecture of adaptation

Consider now an additive quantitative trait with L biallelic loci of the form (S1.3) in mutation-selection-drift balance. Selection can be either directional (towards an optimum $Z_{\mathrm{opt}}(t<0) = Z^0_{\mathrm{opt}}$ at or outside the edges of the phenotype range) or stabilizing towards an intermediate optimum $0 < Z^0_{\mathrm{opt}} < \gamma L$. After an environmental change, the optimum shifts to a new value $Z_{\mathrm{opt}}(t\geq 0) > Z^0_{\mathrm{opt}}$. All loci have equal effect γ and we assume that $Z^0_{\mathrm{opt}} := (L-d)\gamma$ can be matched by an appropriate genotype (*i.e.*, in a monomorphic population), with $d \leq L$ loci carrying the a_i allele and $L-d$ loci carrying the A_i allele. The case $d = L$ corresponds to pure directional selection in the old environment and $0 < d < L$ to stabilizing selection. Mutation rates are sufficiently low that allele frequency distributions in the standing genetic variation (SGV) are U-shaped (concentrated close to $p_i = 0$ and 1). Without restriction, we assume that in the old environment the frequencies p_i of A_i alleles at the first d loci are close to 0, while at the remaining $L-d$ loci the frequencies $q_i = 1 - p_i$ of a_i alleles are close to 0.

We track the adaptive process not as a function of time, but of the mean trait $\bar{Z}$ and sample the population when it has reached a threshold value $\bar{Z} = Z^0_{\mathrm{opt}}+c_Z\gamma \leq Z_{\mathrm{opt}}(t\geq 0)$, where c_Z measures the distance to Z^0_{opt} at which the adaptive architecture is recorded in units of "mutational step-sizes". Adaptive progress of the mean trait towards the sampling point results from two processes: the increase in the frequency p_i of beneficial alleles A_i at the first d loci and the decrease in the frequency q_j of deleterious mutants a_j at the remaining loci. At the sampling threshold, we have

$$\sum_{i=1}^{d} p_i - \sum_{j=d+1}^{L} q_j = c_Z, \tag{S1.20}$$

where the first sum accounts for the contribution of beneficial A_i alleles and the second sum for the (negative) contribution of deleterious a_j mutants. We can derive the joint allele-frequency distribution (the adaptive architecture in the sense of Barghi et al. 2020) under the following assumptions.

Only beneficial mutations

For a wide range of parameter space, the contribution of deleterious alleles to the threshold condition (S1.20) is negligible ($q_j \approx 0$) and the non-trivial part of the adaptive architecture is entirely made up of beneficial alleles. In this case, (S1.20) reduces to (S1.15) and the adaptive architecture is directly given by (S1.18). Obviously, this is always the case if the trait is initially under directional selection. For stabilizing selection, the approximation assumes that deleterious mutations in the "wrong" direction have largely been eliminated when the sampling threshold is reached. For this to be accurate, the sampling distance $c_Z\gamma$ must be much larger than the total effect of deleterious alleles in the SGV, *i.e.*, either c_Z is large or levels of SGV for deleterious alleles are low.

For Gaussian stabilizing selection (S1.2) and equal mutation rates ($\mu_i \equiv \mu$, $\Theta_i \equiv \Theta$), in particular, the average frequency of a deleterious allele in the SGV is $q_j \approx 2\mu/(\sigma\gamma^2)$ (house-of-cards approximation, *cf.* Bürger, 2000) and accordingly their summed contribution towards the trait reads $2(L-d)\mu/(\sigma\gamma)$. Since frequency shifts of deleterious alleles will contribute to adaptation at most proportional to the number of deleterious loci, this effect should be compared with $\gamma c_Z(L-d)/L$, leading to the condition

$$\mu \ll \frac{\sigma\gamma^2 c_Z}{2L}, \quad \textit{i.e.}, \quad \Theta \ll \frac{S c_Z}{L} \tag{S1.21}$$

with $\Theta = 2N_e\mu$ and $S = N_e\sigma\gamma^2$ ($\equiv S_i$).

Remark 2 In terms of the background mutation parameter $\Theta_{bg} = \Theta(d-1)$, the condition (S1.21) can be rewritten as

$$c_Z \gg \frac{\Theta_{bg}}{S(d-1)/L}$$

for the adaptation distance at which the deleterious variation becomes irrelevant. For example, $L = 10$ and $d = 5$, as in Fig. 3 of the *main text*, produces a threshold value of $\Theta_{bg}/4$ for $S = 10$ (and $\Theta_{bg}/40$ for $S = 100$). In this figure, we see that for sampling at $c_Z = 1$, deleterious variation starts to play a role at $\Theta_{bg} = 1$ and becomes substantial at $\Theta_{bg} = 10$ for $S = 10$ (resp. Θ_{bg} between 10 and 100 for $S = 100$), which is in line with this rough prediction.

Marginal distributions of ordered allele frequencies While the joint allele frequency distribution (S1.18) gives the most comprehensive description of the adaptive architecture, it is a high-dimensional object. In our figures, we display marginal distributions for single loci, which generally requires $(d-2)$-fold integration. A numerical method to perform these integrals is described in the *Computational Supplement* (deposited on *Dryad*, Höllinger et al., 2023). For equal loci ($\gamma_i \equiv \gamma$, $\Theta_i \equiv \Theta$), also their marginal distributions are identical. To capture more information from the joint distribution, we therefore order all loci according to their contribution to the adaptive response (for equal locus effects this means: according to the frequency of the adaptive allele) and

derive marginal distributions for these size-ordered loci. This requires appropriate restrictions of the integration range. For example, for the marginal distribution of the major locus (with the largest allele frequency $p_>$) out of three beneficial loci, we require $1 \geq p_> \geq p_< \geq c_Z - p_> - p_< \geq 0$, where $p_<$ is the second largest frequency of a beneficial allele. For equal mutation rate Θ at all loci, the marginal distribution derives as

$$\mathrm{P}_{\max}[p_>|\Theta, c_Z] = 6 \int_{\frac{c_Z - p_>}{2}}^{c_Z - p_>} \Big(\mathrm{P}[\{p_>, p_<, c_Z - p_> - p_<\}|\Theta, c_Z] \cdot \mathrm{H}[p_> - p_<] \cdot \mathrm{H}[c_Z - p_> - p_<] \Big) dp_< \tag{S1.22}$$

with Heaviside-functions $\mathrm{H}[x] = 1$ if $x \geq 0$ and $\mathrm{H}[x] = 0$ else. The factor 6 accounts for the conditioning on the order of allele frequencies at the three loci. Further integrals that are used for the figures of the main text are provided in the *Computational Supplement* (Höllinger et al., 2023).

Non-trivial contribution of deleterious mutations

With high levels of SGV and/or low sampling distance γc_Z, the contribution by deleterious mutations cannot be neglected. For equal locus effects and Gaussian selection (or any symmetric scheme of stabilizing selection), however, we can extend our formalism to obtain analytical results. The key observation is that prior to the environmental change, all mutations at all loci have the same (expected) fitness effect and can therefore be treated as equivalent. While this is no longer the case after the environmental change, we can still use the Yule process approximation under the assumption that virtually all adaptation occurs from the SGV and drift and new mutation during the adaptive phase can be ignored. Note that this is exactly the parameter range where deleterious variation will likely make an important contribution. We then proceed as follows to derive the joint distribution across beneficial and deleterious loci.

1. We define odds for mutant alleles in both directions. While for the first d loci, the $u_i = p_i/(1 - p_i)$ denote odds for A_i mutants, we define parameters $u_j = q_j/(1 - q_j)$ as odds of the a_j mutants for loci $d+1$ to L. In the SGV, odds ratios $u_i(0)/u_1(0)$ of mutant allele frequencies at *all* L loci then follow an inverted Dirichlet distribution (as long as $p_i(0), q_j(0) \ll 1$). We also need a measure of the total amount of SGV,

$$\sum_{i=1}^{L} u_i(0) = C_{\mathrm{SGV}}.$$

2. After the environmental change, odds ratios u_i/u_j among two beneficial loci (or among two deleterious loci) remain constant during the deterministic phase of rapid adaptation. Eq. (S1.14) also shows that *products* $u_i u_j$ of odds at one beneficial and one deleterious locus remain constant. We define

$$u_i := \kappa u_i(0),\ 1 \leq i \leq d \quad \text{and} \quad u_j := \kappa^{-1} u_j(0),\ d+1 \leq j \leq L$$

for odds u_i and u_j at the time of sampling at beneficial and deleterious loci, respectively. Using

$$\frac{1}{\kappa}\sum_{i=1}^{d} u_i + \kappa \sum_{j=d+1}^{L} u_j = C_{\mathrm{SGV}},$$

we derive

$$\kappa = \frac{1}{2U_d}\Big(C_{\mathrm{SGV}} \pm \sqrt{C_{\mathrm{SGV}}^2 - 4U_bU_d}\Big),$$

where

$$U_b := \sum_{i=1}^{d} u_i = \sum_{i=1}^{d} \frac{p_i}{1-p_i}\,, \quad U_d := \sum_{j=d+1}^{L} u_j = \sum_{j=d+1}^{L} \frac{q_j}{1-q_j}.$$

We then obtain the joint distribution of allele frequencies at the time of sampling from inverted-Dirichlet-distributed odds ratios in the SGV via a parameter transformation for both beneficial loci (u_i) and deleterious loci (u_j),

$$x_i := \frac{u_i(0)}{u_1(0)} = \frac{u_i}{u_1} = \frac{p_i(1-p_1)}{(1-p_i)p_1}\,, \quad x_j := \frac{u_j(0)}{u_1(0)} = \frac{\kappa^2 u_j}{u_1} = \kappa^2\,\frac{q_j(1-p_1)}{(1-q_j)p_1},$$

where κ is given above, and we use the sampling condition (S1.20) to express p_1 as

$$p_1 = c_{\mathrm{Z}} + \sum_{j=d+1}^{L} q_j - \sum_{i=2}^{d} p_i\,.$$

3. We then can proceed as above and define a Jacobian matrix for the parameter transformation. In practice, we rather derive marginal distributions via Monte-Carlo integration, as described in the *Computational Supplement* (Höllinger et al., 2023, on *Dryad*). In contrast to the case of only beneficial variation, the transformation depends (via κ) on C_{SGV} as an additional parameter, measuring the total amount of SGV. Typically, C_{SGV} depends on mutation rates *and* selection strength in the ancestral environment. This makes the adaptive architecture at the time of sampling dependent on selection parameters – albeit *not* selection during the adaptive phase, but selection shaping the SGV.

Remark 3: Scaling When comparing models with a different number of loci L and/or population sizes N_{e}, we need to make a choice how to scale the model parameters, or which quantities to keep constant. Due to the key role of the background mutation rate Θ_{bg} for our results, we fix this parameter in all comparisons. If we start with the same proportion of beneficial *vs.* deleterious loci (*e.g.*, always in the middle of the phenotype range), this approximately corresponds to a stable total mutational input, $L\Theta$, per generation. We also keep the selection strength $S = N_{\mathrm{e}}\sigma\gamma^2$ prior to the environmental change constant. Since $L\Theta/S$ measures the total expected level of SGV (the aggregate mutant frequency across all loci) in the house-of-cards regime, this is also kept constant with this choice. In the context of adaptation from SGV, in particular, this means that

the expected number of competing mutant copies at all background loci *per copy at the focal locus* is held constant.

Note that the strength of directional selection scales $\sim (Z_{\mathrm{opt}} - \bar{Z})$ with this choice, which is $\sim L$ if the size of the shift is proportional to the phenotype range, but constant if we shift Z_{opt} by the same number of mutational steps, independently of L. Since the selection strength after the environmental change does not enter in our analytical results, both choices are possible.

S1.3 Proofs

Here, we summarize the proofs from this supplement.

Proof of Theorem 1 We can write

$$\begin{aligned}\bar{w}_{A_i} = \langle w(Z + (1-\eta_i)\gamma_i)\rangle &= \bar{w} + \left\langle \sum_{k=1}^{\infty} \frac{(\gamma_i(1-\eta_i))^k}{k!} \sum_{j=k}^{\infty} \frac{j! w_j}{(j-k)!} Z^{j-k} \right\rangle \\ &= \bar{w} + \sum_{k=1}^{\infty} \gamma_i{}^k \sum_{j=k}^{\infty} \binom{j}{k} w_j \left\langle (1-\eta_i) Z^{j-k} \right\rangle ,\end{aligned}$$

where we use $(1-\eta_i)^k = (1-\eta_i)$. Analogously,

$$\bar{w}_{a_i} = \langle w(Z - \eta_i\gamma_i)\rangle = \bar{w} + \sum_{k=1}^{\infty} (-\gamma_i)^k \sum_{j=k}^{\infty} \binom{j}{k} w_j \left\langle \eta_i Z^{j-k} \right\rangle$$

and thus

$$\dot{p}_i = p_i(1-p_i) \sum_{k=1}^{\infty} \gamma_i{}^k \sum_{j=k}^{\infty} \binom{j}{k} w_j \Big(\langle Z^{j-k}\rangle - (1+(-1)^k)\langle \eta_i Z^{j-k}\rangle \Big) \tag{S1.23}$$

Assuming LE, we can express $\langle \eta_i Z^k \rangle$ recursively as

$$
\begin{aligned}
\left\langle \eta_i Z^k \right\rangle &= \sum_{\ell=0}^{k} \binom{k}{\ell} \left\langle \eta_i (\eta_i \gamma_i)^\ell (Z - \eta_i \gamma_i)^{k-\ell} \right\rangle \\
&= p_i \sum_{\ell=0}^{k} \binom{k}{\ell} {\gamma_i}^\ell \left\langle (Z - \eta_i \gamma_i)^{k-\ell} \right\rangle \\
&= p_i \sum_{\ell=0}^{k} \binom{k}{\ell} {\gamma_i}^\ell \Bigg(\langle Z^{k-\ell} \rangle + \sum_{m=1}^{k-\ell} \binom{k-\ell}{m} (-\gamma_i)^m \left\langle \eta_i Z^{k-\ell-m} \right\rangle \Bigg) \\
&= p_i \Bigg(\sum_{\ell=0}^{k} \binom{k}{\ell} {\gamma_i}^\ell \langle Z^{k-\ell} \rangle + \sum_{\ell=0}^{k} \sum_{m=1}^{k-\ell} \binom{k}{\ell} \binom{k-\ell}{m} (-1)^m {\gamma_i}^{\ell+m} \left\langle \eta_i Z^{k-\ell-m} \right\rangle \Bigg) \\
&= p_i \Bigg(\sum_{\ell=0}^{k} \binom{k}{\ell} {\gamma_i}^\ell \langle Z^{k-\ell} \rangle + \sum_{j=1}^{k} \sum_{m=1}^{j} \binom{k}{j} \binom{j}{m} (-1)^m {\gamma_i}^j \left\langle \eta_i Z^{k-j} \right\rangle \Bigg) \\
&= p_i \Bigg(\langle Z^k \rangle + \sum_{\ell=1}^{k} {\gamma_i}^\ell \binom{k}{\ell} \langle (1-\eta_i) Z^{k-\ell} \rangle \Bigg)
\end{aligned}
$$

using $\sum_{m=1}^{j} \binom{j}{m} (-1)^m = -1$. The ansatz

$$
\begin{aligned}
\langle \eta Z^k \rangle &:= p_i \Bigg(\langle Z^k \rangle + \sum_{n=1}^{k} {\gamma_i}^n \binom{k}{n} G_n(p_i) \langle Z^{k-n} \rangle \Bigg) \qquad \text{(S1.24)} \\
&= p_i \Bigg(\langle Z^k \rangle + \sum_{n=1}^{k} {\gamma_i}^n \binom{k}{n} \langle Z^{k-n} \rangle \\
&\quad - \sum_{n=1}^{k} {\gamma_i}^n \binom{k}{n} p_i \Bigg(\langle Z^{k-n} \rangle + \sum_{m=1}^{k-n} {\gamma_i}^m \binom{k-n}{m} G_m(p_i) \langle Z^{k-n-m} \rangle \Bigg) \Bigg),
\end{aligned}
$$

and equating terms of order ${\gamma_i}^n$,

$$
\binom{k}{n} G_n(p_i) = \binom{k}{n} (1-p_i) - p_i \sum_{\ell=1}^{n-1} \binom{k}{\ell} \binom{k-\ell}{n-\ell} G_{n-\ell}(p_i),
$$

then leads to a recursion for $G_n(p_i)$,

$$
G_n(p_i) = 1 - p_i \sum_{\ell=0}^{n-1} \binom{n}{\ell} G_\ell(p_i), \qquad \text{(S1.25)}
$$

for $n \geq 1$, where we set $G_0(p_i) := 1$. We now show the following

Lemma 1.1 The recursion (S1.25) is solved by

$$
G_n(p_i) = \sum_{k=0}^{n} \sum_{\ell=0}^{k} \binom{k}{\ell} (\ell+1)^n (-1)^\ell {p_i}^k \,. \qquad \text{(S1.26)}
$$

Proof of Lemma 1.1 We prove the Lemma 1.1 by induction. We verify that $G_0(p_i) = 1$ and assume that (S1.26) holds for $\ell < n$. Then

$$G_n(p_i) = 1 - p_i \sum_{\ell=0}^{n-1} \binom{n}{\ell} \sum_{k=0}^{\ell} \sum_{j=0}^{k} \binom{k}{j} (j+1)^{\ell} (-1)^j {p_i}^k.$$
$$= 1 - \sum_{k=0}^{n-1} {p_i}^{k+1} \sum_{\ell=k}^{n-1} \binom{n}{\ell} \sum_{j=0}^{k} \binom{k}{j} (j+1)^{\ell} (-1)^j.$$

Using that $\sum_{j=0}^{k} \binom{k}{j} P(j)(-1)^j = 0$ for any polynomial $P(j)$ of degree less than k,

$$\begin{aligned}
G_n(p_i) &= 1 - \sum_{k=0}^{n-1} {p_i}^{k+1} \sum_{\ell=0}^{n-1} \binom{n}{\ell} \sum_{j=0}^{k} \binom{k}{j} (j+1)^{\ell} (-1)^j \\
&= 1 - \sum_{k=0}^{n-1} {p_i}^{k+1} \sum_{j=0}^{k} \binom{k}{j} (-1)^j \sum_{\ell=0}^{n-1} \binom{n}{\ell} (j+1)^{\ell} \\
&= 1 - \sum_{k=0}^{n-1} {p_i}^{k+1} \sum_{j=0}^{k} \binom{k}{j} (-1)^j \Big((j+2)^n - (j+1)^n \Big) \\
&= 1 + \sum_{k=0}^{n-1} {p_i}^{k+1} \left(\sum_{j=0}^{k} \binom{k}{j} (-1)^j (j+1)^n + \sum_{j=1}^{k+1} \binom{k}{j-1} (-1)^j (j+1)^n \right) \\
&= 1 + \sum_{k=0}^{n-1} {p_i}^{k+1} \left(1 + \sum_{j=1}^{k} \left[\binom{k}{j} + \binom{k}{j-1} \right] (-1)^j (j+1)^n + (-1)^{k+1} (k+2)^n \right) \\
&= 1 + \sum_{k=0}^{n-1} {p_i}^{k+1} \left(1 + \sum_{j=1}^{k} \binom{k+1}{j} (-1)^j (j+1)^n + (-1)^{k+1} (k+2)^n \right) \\
&= 1 + \sum_{k=0}^{n-1} {p_i}^{k+1} \sum_{j=0}^{k+1} \binom{k+1}{j} (-1)^j (j+1)^n \\
&= 1 + \sum_{k=1}^{n} {p_i}^{k} \sum_{j=0}^{k} \binom{k}{j} (-1)^j (j+1)^n \\
&= \sum_{k=0}^{n} \sum_{j=0}^{k} \binom{k}{j} (-1)^j (j+1)^n {p_i}^k
\end{aligned}$$

which proves the lemma. To complete the proof of Theorem 1 we insert (S1.24) in

(S1.23),

$$\dot{p}_i = p_i(1-p_i)\sum_{k=1}^{\infty}{\gamma_i}^k\sum_{j=k}^{\infty}\binom{j}{k}w_j\Big(\langle Z^{j-k}\rangle-(1+(-1)^k)p_i\sum_{n=0}^{j-k}{\gamma_i}^n\binom{j-k}{n}G_n(p_i)\langle Z^{j-k-n}\rangle\Big)$$

$$= p_i(1-p_i)\sum_{k=1}^{\infty}\Big({\gamma_i}^k\sum_{j=k}^{\infty}\binom{j}{k}w_j\langle Z^{j-k}\rangle$$

$$-(1+(-1)^k)\sum_{j=k}^{\infty}w_jp_i\sum_{n=0}^{j-k}{\gamma_i}^{k+n}\binom{j}{k}\binom{j-k}{n}G_n(p_i)\langle Z^{j-k-n}\rangle\Big)$$

$$= p_i(1-p_i)\sum_{k=1}^{\infty}\Big({\gamma_i}^k\sum_{j=k}^{\infty}\binom{j}{k}w_j\langle Z^{j-k}\rangle$$

$$-(1+(-1)^k)p_i\sum_{n=0}^{\infty}{\gamma_i}^{k+n}\binom{k+n}{n}G_n(p_i)\sum_{j=k+n}^{\infty}\binom{j}{k+n}w_j\langle Z^{j-k-n}\rangle\Big)$$

$$= p_i(1-p_i)\sum_{k=1}^{\infty}\Big({\gamma_i}^k\,\frac{f^{(k)}(Z)}{k!}-(1+(-1)^k)p_i\sum_{n=0}^{\infty}{\gamma_i}^{k+n}\binom{k+n}{n}G_n(p_i)\frac{f^{(k+n)}(Z)}{(k+n)!}\Big)$$

Ordering this expression as a power series of ${\gamma_i}^m$ results in

$$\dot{p}_i = p_i(1-p_i)\sum_{m=1}^{\infty}{\gamma_i}^m\,\frac{f^{(m)}(Z)}{m!}\Big(1-p_i\sum_{n=0}^{m-1}\binom{m}{n}(1+(-1)^{m-n})G_n(p_i)\Big).$$

Finally,

$$A_m(p_i) := 1-p_i\sum_{n=0}^{m-1}\binom{m}{n}\big(1+(-1)^{n+m}\big)G_n(p_i)$$

$$= 1-p_i\sum_{n=0}^{m-1}\sum_{k=0}^{n}\sum_{\ell=0}^{k}\binom{m}{n}\binom{k}{\ell}(-1)^\ell(\ell+1)^n\big(1+(-1)^{n+m}\big){p_i}^k$$

$$= 1-p_i\sum_{k=0}^{m-1}{p_i}^k\sum_{\ell=0}^{k}\binom{k}{\ell}(-1)^\ell\sum_{n=0}^{m-1}\binom{m}{n}(\ell+1)^n\big(1+(-1)^{n+m}\big)$$

$$= 1-p_i\sum_{k=0}^{m-1}{p_i}^k\sum_{\ell=0}^{k}\binom{k}{\ell}(-1)^\ell\Big((\ell+2)^m-2(\ell+1)^m+\ell^m\Big)$$

$$= 1-p_i\sum_{k=0}^{m-1}{p_i}^k\sum_{\ell=0}^{k+2}\binom{k+2}{\ell}(-1)^\ell\ell^m$$

completing the proof of the theorem.

Proposition 1.1 In addition, we show that $A_m(p_i\!=\!1) = -(-1)^m$.

Proof of Proposition 1.1 We have

$$\begin{aligned}
A_m(p_i\!=\!1) &= 1-\sum_{k=1}^{m-1}\sum_{\ell=0}^{k+1}\binom{k+1}{\ell}(-1)^\ell \ell^m \\
&= 1-\left(1+\sum_{\ell=0}^{m}\ell^m(-1)^\ell\sum_{j=0}^{m-\ell}\binom{\ell+j}{\ell}\right) \\
&= -\sum_{\ell=0}^{m}\ell^m(-1)^\ell\binom{m+1}{\ell+1} \\
&= \sum_{\ell=1}^{m+1}(\ell-1)^m(-1)^\ell\binom{m+1}{\ell} \\
&= -(0-1)^m(-1)^0\binom{m+1}{0} = -(-1)^m,
\end{aligned}$$

where we use again that $\sum_{j=0}^{k}\binom{k}{j}P(j)(-1)^j = 0$ for any polynomial $P(j)$ of degree less than k.

References

Barghi, N., Hermisson, J., and Schlötterer, C. (2020). Polygenic adaptation: a unifying framework to understand positive selection. *Nature Reviews Genetics*, 21(12):769–781.

Barton, N. H. (1986). The maintenance of polygenic variation through a balance between mutation and stabilizing selection. *Genetical Research*, 47(3):209–216.

Bürger, R. (2000). *The mathematical theory of selection, recombination, and mutation.* Wiley, Chichester, UK.

Falconer, D. and Mackay, T. (1996). *Introduction to Quantitative Genetics.* Longmans Green, Harlwo, Essex, UK, 4th edition.

Hermisson, J. and Pennings, P. S. (2005). Soft sweeps. *Genetics*, 169(4):2335–2352.

Hermisson, J. and Pfaffelhuber, P. (2008). The pattern of genetic hitchhiking under recurrent mutation. *Electronic Journal of Probability*, 13:2069–2106.

Höllinger, I., Pennings, P. S., and Hermisson, J. (2019). Polygenic adaptation: From sweeps to subtle frequency shifts. *PLoS Genet*, 15(3):e1008035.

Höllinger, I., Wölfl, B., and Hermisson, J. (2023). A theory of oligogenic adaptation of a quantitative trait, Dryad, Dataset. https://doi.org/10.5061/dryad.573n5tbc9.

Jain, K. and Stephan, W. (2017). Rapid adaptation of a polygenic trait after a sudden environmental shift. *Genetics*, 206(1):389–406.

Joyce, P. and Tavaré, S. (1987). Cycles, permutations and the structure of the yule process with immigration. *Stochastic processes and their applications*, 25:309–314.

Moran, P. A. P. (1958). Random processes in genetics. *Mathematical Proceedings of the Cambridge Philosophical Society*, 54(1):60–71.
